# Draft Genome Sequence of *Lactobacillus fermentum* Strain 3872

**DOI:** 10.1128/genomeA.01006-13

**Published:** 2013-11-27

**Authors:** Andrey V. Karlyshev, Kavita Raju, Vyacheslav M. Abramov

**Affiliations:** School of Life Sciences, Faculty of Science, Engineering and Computing, Kingston University, Kingston upon Thames, United Kingdoma; Institute of Immunological Engineering, Lyubuchany, Moscow Region, Russiab

## Abstract

This report describes a draft genome sequence of *Lactobacillus fermentum* strain 3872. The data revealed remarkable similarity to and dissimilarity with the published genome sequences of other strains of the species. The absence of and variation in structures of some adhesins and the presence of an additional adhesin may reflect adaptation of the bacterium to different host systems and may contribute to specific properties of this strain as a new probiotic.

## GENOME ANNOUNCEMENT

*Lactobacillus fermentum* isolated from various sources, including humans, poultry, and pigs, was found to be beneficial as a probiotic (1). Application of *L. fermentum* affects body composition and metabolism, providing beneficial effects on human health (2). This bacterium has been used for treatment of gastrointestinal and respiratory illnesses (3). This study describes a draft genome sequence of *L. fermentum* strain 3872, isolated from breast milk from a healthy woman in Russia in 2011. This draft genome sequence is remarkably different from the complete genomes sequences of the other strains of this species (CECT 5716, IFO 3956, and F-6) available at the time of preparation of this article for publication. Despite the presence of highly conserved regions and almost identical rRNA genes, only 70% of reads could be mapped onto the reference genome sequences.

Sequencing using IonTorrent PGM and Chip 314 produced 414,245 reads, which were assembled using the IonTorrent assembler into 284 contigs over 1 kb in size, with a maximum length of 76,656 bases. The total size of the assembly (2.21 Mb, with 29.46-fold genome coverage) and GC content (50.9%) are in good agreement with figures for the reference strains (2.06 to 2.10 Mb and 51.5 to 51.7% for size and GC content ranges, respectively). Genome sequence annotation was conducted using the GenBank microbial genome annotation pipeline (NCBI) and RAST (4), which predicted 2,087 protein-encoding sequences (with the largest encoded protein being an ATP-dependent helicase, at 1,337 amino acids) and 2,060 protein-encoding sequences (with proteins of up to 1,447 amino acids long, for DNA polymerase III).

The test and reference genomes share a gene encoding colicin V (5), which may contribute to the probiotic properties of these bacteria. There are general multidrug resistance genes (e.g., encoding efflux pump proteins) and genes responsible for resistance to arsenate, heavy metals, tetracycline, beta-lactam antibiotics, and fluoroquinolones. There is a large number of transposable and insertion sequence (IS) elements (e.g., several copies of the IS*200* transposase gene).

All strains also contain highly similar copies of a gene encoding a fibronectin-binding protein (up to 99% identity over the entire lengths of the proteins). Only a short C-terminal region of one mucus-binding protein (MBP) found in all reference strains is present in the test strain, with another MBP completely missing from the latter. One outstanding feature of the genome of *L. fermentum* strain 3872 is the presence of a collagen-binding protein (CBP)-encoding gene completely missing from all three reference strains. This large (1,055-amino-acid-long) CBP has a number of long repeats in the C-terminal area and has the maximum level of similarity (up to 87% identity) to CBPs found in *L. paracasei*, *L. oris*, and *L. casei*. Compared to other CBPs, this protein of *L. fermentum* 3872 is unique due to the presence of an additional copy of a repeated element (ca. 80 amino acids in size), which may be responsible for binding to host cell receptors. The strain-to-strain variation in a repertoire of *L. fermentum* adhesins may reflect adaptation of this microorganism to specific host systems.

### Nucleotide sequence accession numbers.

This whole-genome shotgun project has been deposited at DDBJ/EMBL/GenBank under the accession number AVCT00000000. The version described in this paper is version AVCT01000000.
